# Synthesis and Biocompatibility Evaluation of PCL Electrospun Membranes Coated with MTA/HA for Potential Application in Dental Pulp Capping

**DOI:** 10.3390/polym14224862

**Published:** 2022-11-11

**Authors:** Soumya Sheela, Fatma Mousa AlGhalban, Khalil Abdelrazek Khalil, Tahar Laoui, Vellore Kannan Gopinath

**Affiliations:** 1 Sharjah Institute for Medical Research, University of Sharjah, Sharjah 27272, United Arab Emirates; 2 Department of Mechanical & Nuclear Engineering, College of Engineering, University of Sharjah, Sharjah 27272, United Arab Emirates; 3 Department of Preventive and Restorative Dentistry, College of Dental Medicine, University of Sharjah, Sharjah 27272, United Arab Emirates

**Keywords:** polycaprolactone, human dental pulp stem cells, pulp capping, hydroxyapatite, mineral trioxide aggregate, electrospinning

## Abstract

This study aimed to develop polycaprolactone (PCL) electrospun membranes coated with mineral trioxide aggregate/hydroxyapatite (MTA/HA) as a potential material for dental pulp capping. Initially, the PCL membrane was prepared by an electrospinning process, which was further surface coated with MTA (labeled as PCLMTA) and HA (labeled as PCLHA). The physico-chemical characterization of the fabricated membranes was carried out using field emission scanning electron microscopy (FE-SEM)/Energy dispersive X-ray (EDX), X-ray diffraction (XRD), Raman spectroscopy, and contact angle analysis. The biocompatibility of the human dental pulp stem cells (hDPSCs) on the fabricated membranes was checked by XTT assay, and the hDPSCs adhesion and spreading were assessed by FE-SEM and confocal microscopy. The wound healing ability of hDPSCs in response to different electrospun membrane extracts was examined by scratch assay. The surface morphology analysis of the membranes by FE-SEM demonstrated a uniform nanofibrous texture with an average fiber diameter of 594 ± 124 nm for PCL, 517 ± 159 nm for PCLHA, and 490 ± 162 nm for PCLMTA. The elemental analysis of the PCLHA membrane indicated the presence of calcium and phosphorous elements related to HA, whereas the PCLMTA membrane showed the presence of calcium and silicate, related to MTA. The presence of MTA and HA in the PCL membranes was also confirmed by Raman spectroscopy. The water contact analysis demonstrated the hydrophobic nature of the membranes. The results indicated that PCL, PCLHA, and PCLMTA membranes were biocompatible, while PCLMTA exhibited better cell adhesion, spreading, and migration.

## 1. Introduction

Dental caries is a common health problem in children and adolescents globally and in the United Arab Emirates (UAE) [1,2]. This oral disease is attributed to a cariogenic diet, poor dental hygiene, and oral bacterial colonization resulting in acid production, leading to the demineralization of enamel and tooth cavities. Depending on the severity of the infection, these cavities can be large, sometimes reaching the pulp chamber. The dental pulp is a vital soft tissue in the middle of teeth and is a rich source of stem cells, blood vessels, and nerves; it has many crucial functions, including reparative dentin formation [3]. Odontogenic infections result in progressive damage of the pulp, wherein the only possible way to save the tooth could be root canal treatment; however, when the entire pulp tissue is extirpated in root canal treatment, the tooth loses its vitality. Hence, protecting the exposed vital pulp tissue from further bacterial infection and mechanical injury is essential [4]. 

Pulp capping, whether direct or indirect, helps in pulp regeneration, thereby preserving the vitality and functions of the pulp tissue [5]. In direct pulp capping, a biocompatible material is placed over the exposed pulp tissue, overlaid by suitable restorative material to reestablish function and aesthetics [6]. Clinicians have preferred calcium hydroxide as a gold standard material for direct pulp capping [7]. More recently, mineral trioxide aggregate (MTA) has been introduced as a replacement for calcium hydroxide, which comprises calcium, silicate, and bismuth ions [8,9]. Unlike the high alkalinity and cytotoxicity associated with calcium hydroxide, extensive laboratory and clinical studies favored the use of MTA due to its excellent biocompatibility and sealing capabilities [10]. Though it offers excellent biocompatibility, a long setting time associated with the initial application emerges as one of the shortcomings related to MTA [11]. In addition, placing fresh mixed MTA directly over exposed pulp may induce necrosis and moderate inflammation of the pulp tissue; however, this effect is not as harmful as pure calcium hydroxide [12]. 

To overcome the inflammation, the use of a fibrous engineered matrix as physical support before the application of MTA as a pulp-capping agent has been investigated in many studies [13,14]; however, placing the PCL (polycaprolactone) membrane alone on the exposed pulp could limit the dental stem cells’ proliferation and differentiation ability due to its bio-inert nature [15,16]. Hence, to improve the bioactivity of PCL, different bioactive compounds including hydroxyapatite (HA) [17], bioactive glass [18], and MTA [13] have been incorporated into the PCL solution before the electrospinning process. Several studies have shown that these membranes demonstrated better bioactivity than bare PCL membranes [19,20]. 

Unlike HA, very few studies have been reported on MTA-incorporated PCL electrospun membranes. Most of the studies used PCL and MTA separately, not as a composite membrane [13,14]. There is a recent study on the incorporation of MTA cement with PCL and chitosan, using lyophilization as the method of scaffold preparation, not electrospinning [21]. In pre-clinical studies performed using PCL/MTA electrospun membranes, PCL was placed over the exposed pulp and MTA was applied as a paste over it [14]. To our knowledge, no in vitro studies are reported on PCL electrospun membranes incorporated with MTA. Considering the odontogenic potential reported with MTA [22], incorporating it in the PCL electrospun membrane could offer a better scaffold that could stimulate dental pulp stem cells to differentiate into odontoblasts. 

Hence, this present study aims to develop a PCL electrospun membrane coated with commercially available MTA cement and evaluate, in vitro, its cytotoxicity and cell adhesion behavior. A comparative analysis is also made with PCL membrane coated with HA as well as with bare PCL. 

## 2. Materials and Methods

### 2.1. Fiber Preparation by Electrospinning 

**Figure 1:**
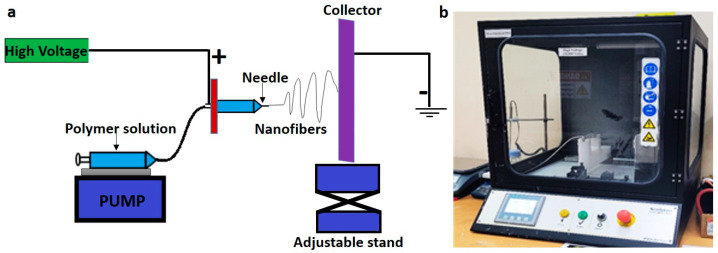
A schematic of the electrospinning setup (**a**), and electrospinning unit in the lab (**b**).

A 10 wt.% of PCL (80,000 MW; Sigma-Aldrich, St. Louis, MO, USA) was prepared using a solvent composed of tetrahydrofuran anhydrous (THF) (Sigma-Aldrich, St. Louis, MO, USA) and dimethylformamide (DMF) (Sigma-Aldrich, St. Louis, MO, USA) in a 1:1 ratio. The solution was then placed on a magnetic stirrer in a sealed glass container overnight to dissolve the PCL pellets completely. Once the polymer pellets were entirely dissolved, the solution was introduced to the electrospinning machine (Nanospinner, Inovenso, Istanbul, Turkey). The samples were fed to the machine in a 10 mL plastic syringe equipped with a 21 G stainless steel needle and ejected at a flow rate of 0.3 mL/h. The distance between the rotating collector and needle was fixed at 14 cm. The electrospinning voltage was set at 18 kV at ambient temperature. The electrospun fibers were collected on an aluminum foil that was placed on a rotating drum with rotation speeds of 250 rpm. The main function of the rotating drum collector was to work as a ground platform as well as to collect the fibers along a wide area to form a homogenous mat. The fibers are not necessary to be aligned because alignment needs another arrangement such as a collector composed of two conductive strips separated by an insulating gap of variable width. A schematic of the electrospinning setup is shown in Figure 1. The as-spun fibers were further divided into three parts: one part was soaked overnight in mineral trioxide aggregate (ProRoot^®^ MTA powder, DENTSPLY Tulsa Dental Specialities, Tulsa, OK, USA, LOT # 240941) solution (1 mg/mL in ethanol); another part was soaked in hydroxyapatite (HA, Fluka Chemie AG. CH-9470 Buchs, Switzerland) solution (1 mg/mL in ethanol); the third part was kept blank. The different ceramic-coated fibers were dried at room temperature before proceeding with the physicochemical characterization.

### 2.2. Field Emission Scanning Electron Microscopy (FE-SEM)/Energy Dispersive X-ray (EDX) Analysis

The electrospun membranes were examined using an FE-SEM microscope (Apreo 2, Thermo Scientific, Waltham, MA, USA) to evaluate their surface morphology. The samples were sputter coated with gold-palladium (Quorum Technologies Ltd, Q150T S plus, Lewes, UK) for conductivity purposes before analysis. The samples were captured at different magnifications at an acceleration voltage of 20 kV. Elemental analysis of the different electrospun membranes was performed using the EDX attachment of the SEM (Oxford Instruments Ultim Max 100, Abingdon, UK). 

### 2.3. X-ray Diffraction Analysis (XRD)

The X-ray diffraction study was carried out on produced fibers using an X-ray diffractometer (D8 Advance, Bruker, Billerica, MA, USA) with Cu Kα radiation (λ = 1.54 Å). The equipment was operated at a scanning rate of 0.5 steps per second and a scanning speed of 0.3°/step.

### 2.4. Raman Spectroscopy 

The different electrospun membranes were examined using a Raman microscope (Renishaw inVia Raman Microscope, Wotton-under-Edge, UK) to check the presence or absence of MTA/HA on the coated fibers. The spectrum range was 100–3500 cm^−1^.

### 2.5. Contact Angle Measurement

The wettability of the electrospun PCL, PCLMTA, and PCLHA samples was measured using a static contact angle analyzer (Rame-hart instrument Co., 21AC, Succasunna, NJ, USA). A deionized water drop of around 3 µL was dropped on dried samples using a micro tip and the contact angle formed on each sample was analyzed and calculated. Each sample was measured three times and the average is reported. 

### 2.6. Stability of the Coating

A one-month degradation study was carried out to check the ceramics coating (MTA and HA) stability. The membranes were cut into 1 cm × 1 cm and were soaked in 1 mL phosphate-buffered saline (PBS) buffer. Samples (n = 3) were then kept in a rocking platform (BENCHROCKER™ 2D, Benchmark Scientific, Sayreville, NJ, USA) with continuous shaking for one month at room temperature. After the incubation, the membranes were washed in PBS and dried at 37 °C, and the surface morphology and elemental analysis were carried out in an FE-SEM coupled with EDX. 

### 2.7. Human Dental Pulp Stem Cells (hDPSCs) Culture

Human dental pulp stem cells were used to evaluate the cytotoxicity of the prepared electrospun PCL membranes. The hDPSCs were procured from CLS cell line services GmbH, Eppelheim, Germany, and the cells were maintained in DMEM/F-12 medium supplemented with 10% fetal bovine serum and 1% penicillin-streptomycin. All the cell culture consumables were obtained from Sigma-Aldrich, St. Louis, MO, USA. The cells from passages 3–6 were used for the study.

### 2.8. Cell Viability Studies on PCL Membranes

For the cell viability analysis, both direct and indirect cell culture methods were utilized following the ISO 10993-5 standards (28). For the direct process, the hDPSCs were directly seeded over the PCL membranes, and the cytotoxic behavior of the cells when in direct contact was evaluated by XTT Assay (Cell Proliferation Kit II, Sigma-Aldrich, St. Louis, MO, USA). The electrospun membranes (PCL, PCLMTA, and PCLHA) were sterilized by UV for one hour before the start of the cell culture experiments. The hDPSCs were seeded at a density of 1 × 10^4^/cm^2^ on top of the materials and were incubated for 24 h and 48 h. For the indirect method, the respective electrospun PCL membranes were incubated with a cell culture medium for 24 h at 37 °C with mild shaking (Incu-Shaker Mini Shaking Incubator, Benchmark Scientific, Sayreville, NJ, USA) and the extracted conditioned medium was treated with the cells. The conditioned medium was added to a monolayer of cells cultured on a 96-well plate at a cell seeding density of 1 × 104 cells per well and was incubated for 24 h and 48 h. XTT assay was carried out after each incubation period following the manufacturer’s instruction. Briefly, samples were washed three times with PBS for the direct method and an adequate amount of XTT solution was added to cover the membrane entirely, which was incubated for four hours. For the indirect method, 50 µL of XTT was layered over the cells and incubated for four hours. The absorbance of the orange-colored formazan product formed was measured at 450 nm and 630 nm in a microplate reader (Synergy H1 microplate Reader, Biotek Instruments, Winooski, VT, USA). 

### 2.9. Cell Adhesion and Spreading Studies by Field Emission Scanning Electron Microscopy (FE-SEM) and Confocal Microscopy 

The adhesion of hDPSCs on the different electrospun membranes was analyzed by FE-SEM and confocal microscopic studies. The hDPSCs at a density of 1 × 10^4^ cells/cm^2^ were seeded on each of the membranes and kept for 48 h incubation. After the incubation period, the cells were analyzed for their spreading behavior by FE-SEM and a confocal microscope. FE-SEM analysis was executed on the samples after fixation for one hour at room temperature with 2.5% (*v*/*v*) glutaraldehyde (Sigma-Aldrich, St. Louis, MO, USA) prepared in phosphate-buffered saline (PBS). The samples were thoroughly rinsed with PBS and subsequently dehydrated in ascending alcohol series (50%, 70%, 90%, and 100%, in each solution for ten minutes) and sputter coating with gold-palladium for FE-SEM analysis.

For the confocal microscopic studies, the membranes were washed three times with PBS and fixed in 4% paraformaldehyde (Sigma-Aldrich, St. Louis, MO, USA) for 20 min at room temperature. The fixed cells were permeabilized with 0.1% Triton X-100 (Sigma-Aldrich, St. Louis, MO, USA) in PBS for 5 min. The permeabilized cells were stained with Texas Red™-X Phalloidin (Thermo Fisher Scientific, Waltham, MA, USA) for 20 min at room temperature. Subsequently, the cells were stained with 4′,6-diamidino-2-phenylindole (DAPI) (Abcam, Cambridge, UK) and were visualized using confocal laser scanning microscopy (CLSM, Nikon Eclipse Ti-S, Nikon Instruments Inc., Melville, NY, USA). 

### 2.10. Wound Healing Assay-Scratch Test

The in vitro wound healing assay was conducted as per the protocols described (29–31). The hDPSCs were seeded onto a 12-well culture plate at a density of 50,000 cells/well and were incubated at 37 °C in a cell culture incubator with 5% CO_2_ and 95% air for 24 h. A perpendicular scratch was made with a sterile 200 µL pipet tip right in the center of the hDPSC monolayer and the cells were treated with the extracts of the different electrospun PCL membranes. For the control group, a normal tissue culture medium was used. The migration of the cells was monitored at other time points (0 h, 24 h, and 48 h), and the images were captured using an inverted phase contrast microscope (Olympus IX73, Olympus, Tokyo, Japan) at 10× magnification. The images were analyzed, and the rate of wound closure was calculated.

### 2.11. Statistical Analysis 

All results analyzed were from three independent experiments and statistical analysis was performed using GraphPad Prism version 9.0.0 (121) software (GraphPad Software, Inc., San Diego, CA, USA). A two-way ANOVA followed by Tukey’s multiple comparisons was performed to check the statistical significance between different groups. A *p*-value of <0.05 was considered statistically significant. 

## 3. Results

### 3.1. FE-SEM/EDX Analysis 

**Figure 2:**
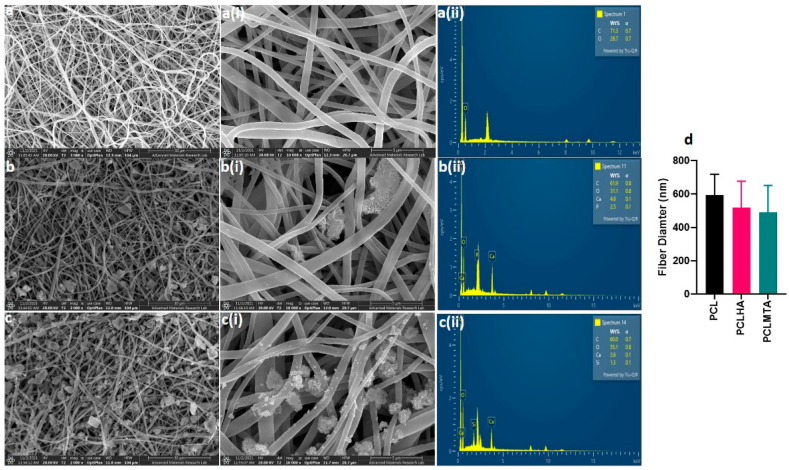
Representative FE-SEM and the corresponding EDX images of the electrospun PCL membranes. FE-SEM images of each electrospun membrane at different magnifications are provided for PCL (**a**,**a**(**i**)), PCLHA (**b**,**b**(**i**)), and PCLMTA (**c**,**c**(**i**)) and the corresponding EDX images for PCL (**a**(**ii**)), PCLHA (**b**(**ii**)), and PCLMTA (**c**(**ii**)). The fiber diameter for each membrane obtained by image J analysis is represented in a bar chart with mean ± standard deviation in (**d**).

The surface morphology and elemental analysis of PCL, PCLHA, and PCLMTA electrospun membranes are represented in Figure 2. The morphological analysis showed that all the electrospun membranes demonstrated almost uniform fiber size with no beads formation. The beadlike structure, shown in Figure 2b,b(i),c,c(i), belongs to HA and MTA particles as can be confirmed by the corresponding EDX images provided (Figure 2b(ii),c(ii)). The average fiber size determined by Image J software (Rasband, W.S, National Institute of Mental Health, Bethesda, MD, USA) analysis was revealed to be 594.53 ± 124.34 nm for PCL, 517.36 ± 159.63 nm for PCLHA, and 490.24 ± 162.41 nm for PCLMTA. The elemental study of the surface of PCLHA showed the respective calcium and phosphorous elements related to hydroxyapatite, confirming the presence of HA in the membrane. The surface elemental analysis of PCLMTA membranes showed the presence of calcium and silicate, which corresponds to MTA. The PCL blank membrane showed the presence of only carbon and oxygen. 

### 3.2. XRD Analysis

**Figure 3:**
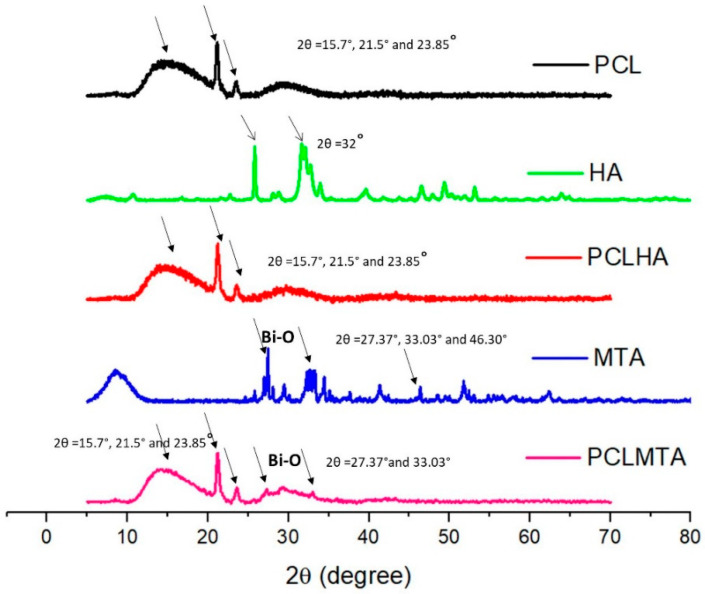
X-ray diffraction (XRD) pattern of PCL, HA, PCLHA, MTA, and PCLMTA. The signature 2θ value of the respective material is represented by arrows in the diffractogram.

Figure 3 represents the XRD analysis of the electrospun PCL membrane and that of bare HA and MTA. The XRD spectrum of PCL shows a semi-crystalline nature, with two sharp peaks at 2θ = 21.5° and 23.85°, along with a broader peak at 2θ = 15.7°. The XRD pattern of HA powder showed two well-resolved peaks at 2θ = 27° and 2θ = 32°. However, in the PCLHA, the corresponding peaks of HA were not prominent, perhaps due to the low concentration of HA used for coating. The MTA powder diffraction pattern demonstrated the corresponding bismuth oxide peaks at 2θ = 27.37°and 33.03°, these peaks were also found in the PCLMTA membrane.

### 3.3. Raman Spectroscopy

**Figure 4:**
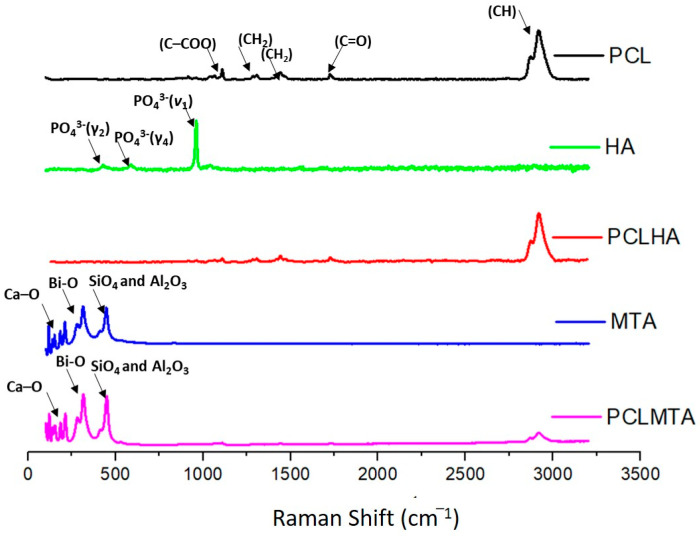
Raman spectroscopic analysis of PCL, HA, PCLHA, MTA, and PCLMTA. The respective Raman shift value of each material is represented by arrows.

The Raman spectral analysis of the different electrospun PCL membranes, HA, and MTA are shown in Figure 4. Some narrow peaks within the range of 900–1100 cm^−1^ (νC–COO, skeletal stretching), 1270–1320 cm^−1^ (ωCH2), 1400–1470 cm^−1^ (δCH2), and 2800–3000 cm^−1^ (νCH) were observed for the PCL membrane. The HA particles showed the characteristics of PO4 vibration bands at 472 cm^−1^, P-O-P bending at 563 and 602 cm^−1^, P-O-P stretching at 960–962 cm^−1^, and P-O-P stretching at 1035–1045 cm^−1^; however, the incorporation of HA in the PCLHA membrane could not be detected.

The Raman spectrum of MTA powder showed Ca-O vibrations near 200–250 cm^−1^. The bismuth oxide peaks were seen at 300 and 450 cm^−1^. The peaks near 400 cm^−1^ could be due to the vibrations of SiO_4_ and Al_2_O_3_. The PCLMTA membrane also showed the peaks mentioned above, which were seen along with the –CH vibrational peak of PCL.

### 3.4. Water Contact Angle

**Figure 5:**
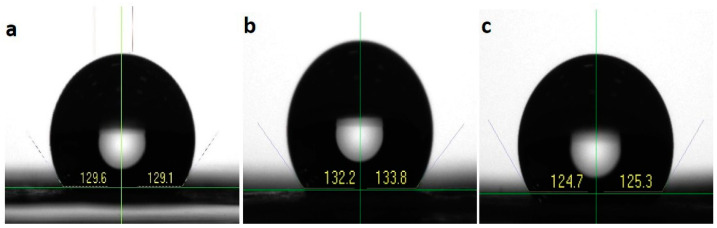
Representative photographs showing the water contact angle for (**a**) PCL, (**b**) PCLHA, and (**c**) PCLMTA membranes.

The hydrophobicity or hydrophilicity of a material can be analyzed by checking the contact angle formed on the material by the water droplet. PCL membranes showed a hydrophobic nature with a contact angle of 129.35 ± 0.35°. The coating of HA and MTA over the PCL membranes also did not significantly vary the hydrophobicity (Figure 5). The average water contact angle calculated for PCLHA and PCLMTA was found to be 129.59 ± 1.131° and 129.03 ± 0.42°, respectively. It is well-known that the application of an HA coating will reduce the contact angle of the matrix; however, due to the low concentration of the MTA and HA (1 mg/mL in ethanol), no change in the hydrophobicity of the PCL matrix was observed. 

### 3.5. HA and MTA Coating Stability

**Figure 6:**
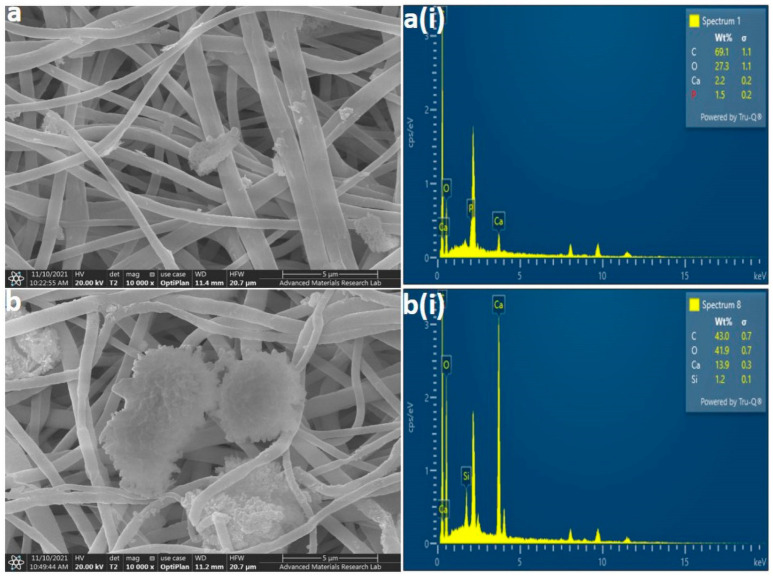
Representative FE-SEM and the corresponding EDX images of electrospun PCLHA (**a**,**a**(**i**)) and PCLMTA (**b**,**b**(**i**)) after one-month degradation study. The nanofibers are intact even after one month and the HA and MTA particles are obvious in the SEM and EDX analysis.

The stability of the HA and MTA coating over the electrospun PCL membranes was checked after a one-month degradation study in PBS. The FE-SEM and EDX analysis showed (Figure 6) that the particles were still embedded on the surface and the nanofibers were intact. The elemental analysis of the particles proved to be calcium and phosphorous for HA and calcium and silicate for MTA.

### 3.6. Cell Viability

**Figure 7:**
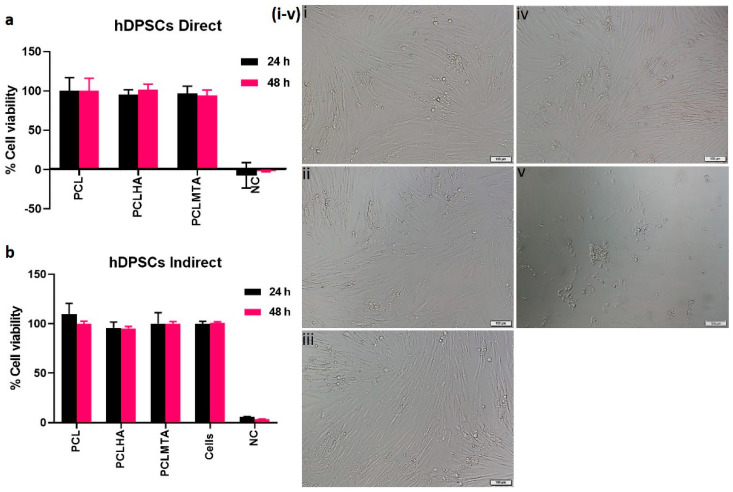
Cell viability analysis of hDPSCs on the electrospun PCL membranes by direct (**a**) and indirect (**b**) XTT method after an incubation period of 48 h. The phase contrast microscopic images of the hDPSCs after 48 h treatment with different PCL-membrane extracts is shown in (**i**–**v**); PCL (**i**), PCLHA (**ii**), and PCLMTA (**iii**). The hDPSCs displayed the same characteristic spindle-shaped morphology as that of the cells in normal cell culture media (**iv**). However, the negative control cells (NC) in DMSO showed rounded morphology (**v**). The scale bar in the figure represents 100 µm.

The XTT analysis of the PCL membranes by direct cytotoxicity method after 24 h and 48 h demonstrated that the tested electrospun membranes were nontoxic. All the electrospun PCL membranes displayed ~100% cell viability even after 48 h of culture (Figure 7a). 

The indirect XTT assay of the test on extract method also showed that the cells grown in the electrospun PCL membranes conditioned media showed good cell viability with respect to the control hDPSCs (Figure 7b). This is also evident from the phase contrast microscopic images of the hDPSCs grown in the presence of PCL membrane extracts (Figure 7i–v). The cells showed the typical spindle-shaped morphology as that of the control hDPSCs. The cells in the DMSO, which was a negative control, indeed showed round morphology.

### 3.7. Cell Adhesion and Spreading Behavior on the Electrospun Membranes

**Figure 8:**
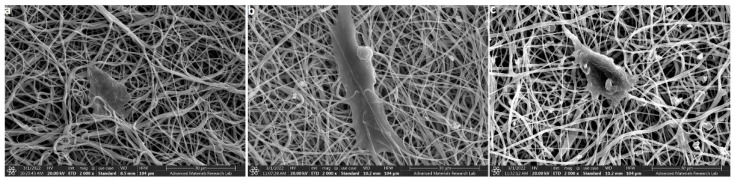
Representative FE-SEM images of the hDPSCs attachment and spreading on (**a**) PCL, (**b**) PCLHA, and (**c**) PCLMTA.

**Figure 9:**
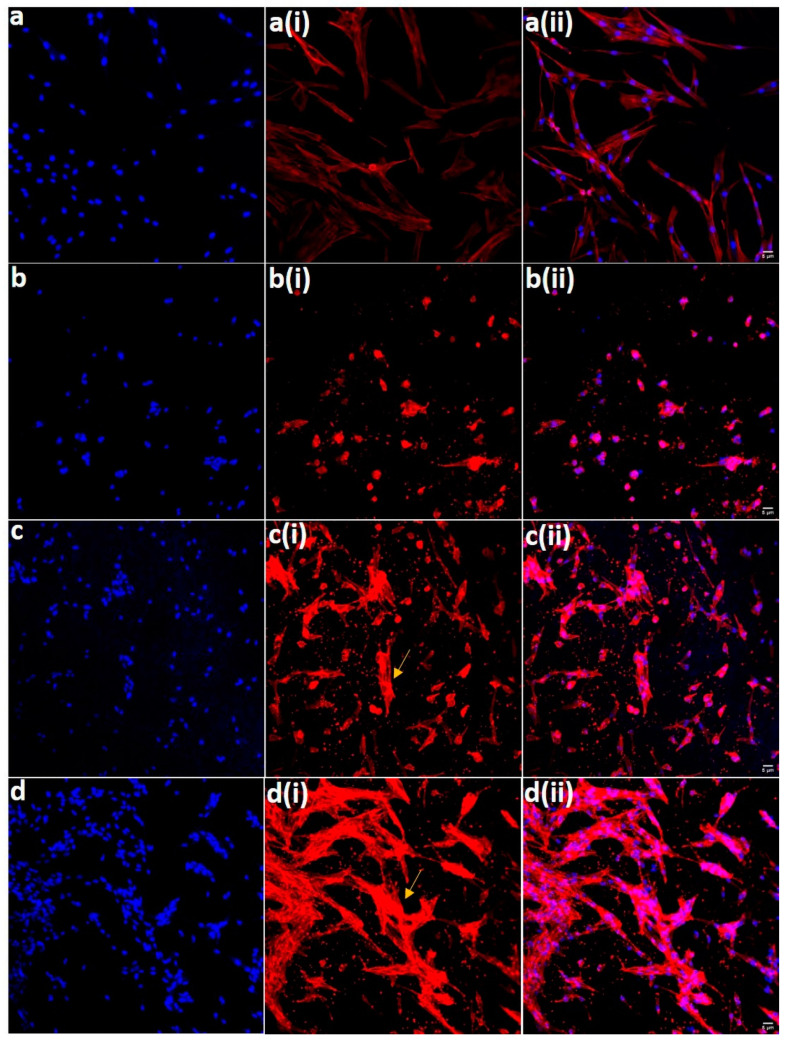
Representative confocal microscopic images of the cytoskeletal arrangement of hDPSCs on (**a**) control coverslips, (**b**) PCL, (**c**) PCLHA, and (**d**) PCLMTA. All the images in the first column represent the nuclear DAPI staining (**blue**), the second column represents the signal from actin (**red**), and the third column represents the merged signals. The spreading and cytoskeletal arrangement of hDPSCs in the PCLHA and PCLMTA are shown by arrows. The scale bar in the figure is 5 μm.

The FE-SEM showing the cell adhesion behavior of the hDPSCs on the electrospun PCL membranes after 48 h incubation is depicted in Figure 8. The images revealed that all the membranes supported cell adhesion, perhaps the good spread morphology of the hDPSCs was observed on PCLHA and PCLMTA electrospun membranes. The adhesion of hDPSCs was further confirmed by confocal imaging (Figure 9). It was observed that the cells showed a spread morphology on all the electrospun membranes except for the bare PCL membrane. The cells adhered to the PCLHA and PCLMTA membranes and demonstrated a well-spread morphology and spindle shape, the same as that of the positive control hDPSCs seeded on the glass coverslips. Filamentous actin fiber organization was evident on the PCLHA and PCLMTA membranes compared to the bare PCL membrane. The cells on the PCL membranes showed a relatively round morphology that could be attributed to the bio-inert nature of the three PCL membranes. Considering PCLHA and PCLMTA, the latter showed comparatively better spreading with significant elongation.

### 3.8. Wound Healing Assay

**Figure 10:**
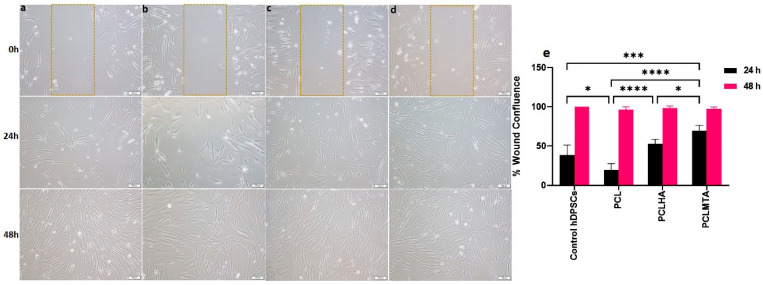
Representative phase contrast microscopic images of the wound healing ability of hDPSCs analyzed for 48 h when treated with different electrospun membrane extracts. The images were shown as: (**a**) control hDPSCs grown on glass coverslips in a normal cell culture medium, (**b**) hDPSCs in PCL membrane extract, (**c**) hDPSCs in PCLHA extract, and (**d**) hDPSCs in PCLMTA extract. The percentage of wound closure was calculated and is represented as a bar chart with mean ± SD (**e**). Statistical significance between the test groups is represented using asterisks; *, *** and **** with the *p* values < 0.05, 0.001, and 0.0001, respectively. The scale bar in the figure is 100 µm.

The wound healing ability of hDPSCs in response to different electrospun membrane extracts was examined by scratch assay. The percentage of wound confluence was calculated with respect to the control hDPSCs grown in cell culture media (Figure 10). The results of the 24 h scratch test showed that the cells seeded on PCLHA and PCLMTA showed a reasonable migration rate compared to the cell group grown in the PCL extract. There was a significant difference in the cell migration observed between PCL vs. PCLHA (**** *p* < 0.0001) and PCL vs. PCLMTA (**** *p* < 0.0001). The latter demonstrated the fastest migration between PCLHA and PCLMTA (* *p* < 0.05). The cells seeded in the standard tissue culture medium showed comparatively better migration rates than bare PCL (* *p* < 0.05) but slower rates than PCLMTA (*** *p* < 0.001). All the cell groups attained 100% wound closure by 48 h.

## 4. Discussion

This study aimed to fabricate a biomaterial that can be utilized for dental pulp capping procedures. Since its inception, electrospinning has been considered a key fabrication technology for preparing nanofibrous membranes, which has gained widespread attention, mainly in tissue regeneration studies [23]. Control over the fine-tuning of surface characteristics such as fiber diameter, porosity, roughness, and thickness makes it an interesting scaffold fabrication technique in various applications [24]. Considering these advantages, we have utilized electrospinning to fabricate nanofibrous membranes in our study. PCL was chosen as a candidate polymeric material for electrospinning due to its high mechanical properties, gradual degradation nature, nontoxic behavior, ease of handling, and nanofiber processing [25]. In addition, PCL was approved by the Food and Drug Administration (FDA) in medical and drug delivery applications owing to its biocompatibility and mechanical properties [26,27]. The processing parameter for the electrospinning was selected based on the literature [28,29], and initial fine-tuning was conducted through trial runs before finalizing the concentration, flow rate, voltage, and solvent concentrations. 

Being a bio-inert polymer, PCL does not have the necessary cues to support and foster the cells and aid in tissue development; hence, MTA and HA were introduced as a coating on the bare PCL membrane to improve its bioactivity [30]. The coating of HA and MTA over PCL membranes was preferred over blending it in the PCL polymer before electrospinning as it would provide adhesion motifs that could be easily accessible for the cells to attach onto and proliferate. The components of ProRoot^®^ MTA include tricalcium silicate, dicalcium silicate, bismuth oxide, tricalcium aluminate, and gypsum [31]. In comparison, HA is the main inorganic component of tooth enamel, dentin, and bone, comprised primarily of calcium and phosphate [32]. The presence of MTA in the PCLMTA membranes was confirmed by physicochemical characterization methods such as EDX, XRD, and Raman spectroscopy, whereas HA presence in the PCLHA membrane was confirmed in EDX. The elemental analysis of the PCLHA membrane showed the presence of calcium and phosphorous and PCLMTA membranes showed the presence of calcium and silicate. The presence of MTA in the PCLMTA was again confirmed by XRD analysis. XRD diffractogram studies showed the corresponding bismuth oxide peaks at 2θ = 27.37°and 33.03° of MTA [33] in the PCLMTA membrane proving the presence of MTA in the membrane after the coating procedure. However, the corresponding diffraction pattern of HA was not so evident in the PCLHA membrane, which could be due to the overlapping of the peaks of PCL with that of HA. The Raman spectral analysis of PCLMTA showed the characteristic Ca-O vibrations near 200–250 cm^−1^, bismuth oxide peaks were seen at 300 and 450 cm^−1^, and vibrational peaks of SiO_4_ and Al_2_O_3_ at 400 cm^−1^ [34]. This substantiates the presence of MTA in the PCL membrane after coating. On the other hand, the high-intensity HA peaks were also not showing for the PCLHA membrane in the Raman spectra. This could be due to low concentration or the physical characteristics of the HA material used in the coating; however, the presence of HA was detected by the SEM and EDX analysis. HA incorporation in the PCL membrane at a concentration of 1 mg/mL could not be detected in XRD and Raman spectroscopic analysis. 

Surface wettability analysis or water contact angle measurement was also performed to check the surface hydrophobicity or hydrophilicity of the electrospun membranes. Measuring the water contact angle formed on the surface of biomaterials is always desirable to check mainly when the material is intended for biomedical applications [35]. It gives a clear picture of the nature of the material surface, whether it is hydrophobic or hydrophilic. The water contact angle measured for PCL showed a hydrophobic range, PCL being well known to be a hydrophobic polymer [36]. The coating of HA and MTA over the PCL membrane also did not demonstrate much change in the contact angle values. Though the coated membranes proved to be hydrophobic, they supported the hDPSCs attachment and spreading compared to bare PCL; this could be due to the bioactivity introduced by the MTA and HA particles present on the PCL membrane [32,37]. 

The in vitro cytotoxicity analysis of the electrospun membranes showed that all the membranes, bare PCL, HA, and MTA-coated PCL membranes, did not induce any toxicity to the human dental pulp stem cells. The results followed other research articles wherein electrospun PCL membranes did not have cytotoxic effects on hDPSCs [38]. However, owing to the bioinert nature of PCL, it lacks the cell adhesion motifs to support cell attachment and spreading. Hence, the addition of bioactive agents or molecules can indeed help in improving cell adhesion behavior [39]. The cell adhesion and spreading behavior of hDPSCs on electrospun PCL, PCLHA, and PCLMTA membranes by SEM and confocal microscopic imaging showed that the cells preferred PCLHA and PCLMTA membranes compared to bare PCL. The cells demonstrated relatively more elongated or spindle morphology in the PCLHA and PCLMTA membranes than bare PCL.

Moreover, the hDPSCs on the PCLHA and PCLMTA membranes demonstrated good actin cytoskeletal arrangement compared to bare PCL. This could be attributed to the presence of HA and MTA embedded within the fibers. MTA is a calcium silicate-based material, and improved cellular response and enhanced production of odontogenic proteins were observed in studies [37,40,41]. Hydroxyapatite, on the other side, is the major inorganic mineral component of bone and is a rich source of calcium and phosphate [42]. Incorporating HA in the matrix could aid in hDPSCs attachment and spreading, which can further enhance the functional activity of the cells to differentiate into odontoblasts. The results observed in the case of PCLHA in our study were in agreement with our previous study wherein the incorporation of nHA in PCL membranes showed improved odontogenic differentiation. The odontogenic-specific genes, such as DSPP and ALP gene expression, were higher in PCLHA than in bare PCL membranes [29]. Between PCLHA and PCLMTA membranes, hDPSCs on the PCLMTA membranes demonstrated more cell adhesion and better cytoskeletal organization compared to PCLHA. Herein our study, HA and MTA provided the necessary bioactivity for the cells, and PCL provided the structural framework such as the biological extracellular matrix with interconnected pores and nano-fibrillar architecture [14]. 

In reparative dentin formation, cellular events such as cell migration, proliferation, and odontogenic differentiation are involved [43]. Wound healing is a crucial aspect to be considered during the deployment of any biomaterial intended for tissue regeneration; especially if the biomaterial is designed to be used as a dental pulp capping agent since the hDPSCs have to attach and migrate, and should result in the initial healing process [44]. Hence, in our study, the potential of the electrospun membranes in the healing process was investigated by an in vitro scratch assay. We could observe that at 24 h, the PCLMTA membranes demonstrated better cell migration compared to the control, PCL, and PCLHA. Similar results were observed in other studies with ProRoot^®^ MTA that favored migrating human bone marrow-derived mesenchymal stem cells and hDPSCs [45,46]. Though the rate of hDPSC migration was less in PCLHA compared to PCLMTA, a significant difference in the rate of cell migration was observed on the PCLHA membrane compared to bare PCL. However, at 48 h of incubation, complete wound healing was observed in all the groups, including the control. The cell migration study also supported the cell attachment and spreading studies wherein the PCLMTA membrane demonstrated better spreading than bare PCL and PCLHA; however, more in vitro and in vivo evaluations are recommended before utilizing these membranes in clinical applications as pulp capping material. In vitro studies such as hemocompatibility and osteo/odontogenic differentiation potential of these tested membranes will be our next focus of study. Since in vitro studies have already confirmed the antimicrobial activity of ProRoot^®^ MTA in the presence of blood [47], therefore we assume that coating fiber meshes with MTA may display antimicrobial activity as well at the site of application; however, this would need further investigation.

## 5. Conclusions

Polycaprolactone (PCL) membrane was prepared using an electrospinning process, followed by coating with mineral trioxide aggregate/hydroxyapatite (MTA/HA). The developed membranes were characterized using a variety of techniques to assess their morphology, and the physicochemical characterization authenticated the nanofibrous structure and presence of calcium, silicate, and bismuth in the PCLMTA membrane, which was found to be cyto-friendly and stable. Although the synthesized membranes were hydrophobic, PCLMTA exhibited a better spreading with spindle-shaped morphology of hDPSCs and enhanced cell migration in the scratch assay compared to PCLHA and bare PCL.

## 6. Limitation of the Study and Future Prospects

Although the presence of HA in PCLHA could be noticed in EDX, its presence was not detected in the XRD and Raman spectroscopy, which can be considered a drawback in this study. Hence, further investigation is warranted on optimizing the concentration of HA while spinning the PCLHA membrane. The future direction of this membrane development work for the purpose of dental pulp capping will have to be directed toward checking the in vitro odontogenic differentiation potential of DPSCs, following which, in vivo pulp capping studies with PCL MTA/HA membranes in simulated animal models are warranted.

## Data Availability

The data presented in this study are available on request from the corresponding author.
